# An unprecedented binuclear cadmium di­thio­carbamate adduct: bis­[μ_2_-*N*-(2-hydroxy­eth­yl)-*N*-iso­propyl­carbamodi­thio­ato-κ^3^ *S*:*S*,*S*′]bis­{[*N*-(2-hydroxy­eth­yl)-*N*-iso­propyl­carbamodi­thio­ato-κ^2^ *S*,*S*′](3-{(1*E*)-[(*E*)-2-(pyridin-3-yl­methyl­idene)hydrazin-1-yl­idene]meth­yl}pyridine-κ*N*)cadmium]} dihydrate

**DOI:** 10.1107/S2056989016012214

**Published:** 2016-08-02

**Authors:** Hadi D. Arman, Pavel Poplaukhin, Edward R. T. Tiekink

**Affiliations:** a Department of Chemistry, The University of Texas at San Antonio, One UTSA Circle, San Antonio, Texas 78249-0698, USA; b Chemical Abstracts Service, 2540 Olentangy River Rd, Columbus, Ohio, 43202, USA; c Centre for Crystalline Materials, Faculty of Science and Technology, Sunway University, 47500 Bandar Sunway, Selangor Darul Ehsan, Malaysia

**Keywords:** crystal structure, cadmium, di­thio­carbamate, hydrogen bonding

## Abstract

The asymmetric unit in the title binuclear compound, [Cd(C_6_H_12_NOS_2_)_2_(C_12_H_10_N_4_)]_2_·2H_2_O, comprises a Cd^II^ atom, two di­thio­carbamate (dtc) anions, a monodentate 3-pyridine­aldazine ligand and a lattice water mol­ecule. The binuclear mol­ecule is constructed by the application of inversion symmetry. One dtc ligand simultaneously chelates one cadmium atom and bridges the centrosymmetric mate, while the other dtc ligand is chelating only. This leads to a centrosymmetric [Cd(dtc)_2_]_2_ core to which are appended two 3-pyridine­aldazine ligands. The resulting NS_5_ donor set is based on an octa­hedron. The three-dimensional mol­ecular packing is sustained by hydroxyl-O—H(hydrox­yl) and water-O—H⋯O(hydrox­yl) hydrogen bonding, leading to supra­molecular layers parallel to (101) which are connected by water-O—H⋯N(pyrid­yl) hydrogen bonding; additional C—H⋯O, S π(chelate ring) inter­actions are also evident. The retention of the central [Cd(dtc)_2_]_2_ core upon adduct formation is unprecedented in the structural chemistry of the zinc-triad di­thio­carbamates.

## Abstract 2

A distorted octa­hedral NS_5_ donor set is found in the binuclear title mol­ecule which features an unprecedented [Cd(di­thio­carbamate)_2_]_2_ core. Mol­ecules are connected into a three-dimensional architecture by O—H⋯O,N hydrogen bonding.

## Chemical context   

The common feature of the structural chemistry of the binary cadmium di­thio­carbamates, *i.e*. mol­ecules of the general formula Cd(S_2_CN*RR*′)_2_ for *R*, *R*′ = alkyl, is the adoption of aggregated species in the solid state. The overwhelming majority of structures are binuclear, [Cd(S_2_CN*RR*′)_2_]_2_, arising from equal numbers of μ_2_-tridentate and bidentate (chelating) ligands (Tiekink, 2003 ▸; Tan, Halim *et al.*, 2016 ▸). The exceptional structures are trinuclear {Cd[S_2_CN(p-tol)furan-2-ylmeth­yl]_2_}_3_ (Kumar *et al.*, 2014 ▸), having two μ_2_-tridentate and four chelating ligands, and one-dimensional polymeric [Cd(S_2_CNMe_2_)_2_]_*n*_ (Bing *et al.*, 2010 ▸), {Cd[S_2_CN(iPr)CH_2_CH_2_OH]_2_}_*n*_ (Tan *et al.*, 2013 ▸; Tan, Halim *et al.*, 2016 ▸) and {Cd[S_2_CN(Me)CH_2_CH(OMe)_2_]_2_}_*n*_ (Ferreira *et al.*, 2016 ▸), having all ligands μ_2_-tridentate. Inter­estingly, supra­molecular isomers were found for the {Cd[S_2_CN(iPr)CH_2_CH_2_OH]_2_}_*n*_ species (Tan *et al.*, 2013 ▸; Tan, Halim *et al.*, 2016 ▸), which were shown to adopt the common binuclear structural motif. Up to now, whenever Cd(S_2_CN*RR*′)_2_ is reacted with bases, *e.g*. pyridyl-donors, the original aggregate is disrupted in that no dtc links are retained between cadmium atoms. Thus, when archetypal, binuclear [Cd(S_2_CNEt_2_)_2_]_2_ (Domenicano *et al.*, 1968 ▸; Dee & Tiekink, 2002 ▸) reacts with monodentate N-donors such as 2,6-di­methyl­pyridine, mononuclear, five-coordinate species result (Lennartson & Håkansson, 2009 ▸). Similarly, bidentate chelating ligands, such as 2,2′-bipyridyl, lead to mononuclear species but with formally six-coordinate cadmium atoms (Airoldi *et al.*, 1990 ▸). Higher nuclearity structures are also formed with bridging, bidentate ligands such as in the one-dimensional coordination polymers formed with μ_2_-1,2-bis­(4-pyrid­yl)ethyl­ene (Chai *et al.*, 2003 ▸) and μ_2_-1,2-bis­(4-pyrid­yl)ethane (Avila *et al.*, 2006 ▸). In the latter structures, six-coordinate, *trans*-N_2_S_4_ donor sets are found. In the present report, crystals of the 1:2 adduct between {Cd[S_2_CN(iPr)CH_2_CH_2_OH)]_2_}_2_ and 3-pyridine­aldazine were isolated and shown by X-ray crystallography that despite having one potentially bidentate bi-pyridyl ligand per Cd[S_2_CN(iPr)CH_2_CH_2_OH)]_2_ unit, the central binuclear core (Tan *et al.*, 2013 ▸; Tan, Halim *et al.*, 2016 ▸) remained intact with the 3-pyridine­aldazine mol­ecules coordinating in a monodentate mode, thereby representing a new structural motif for this class of compound.
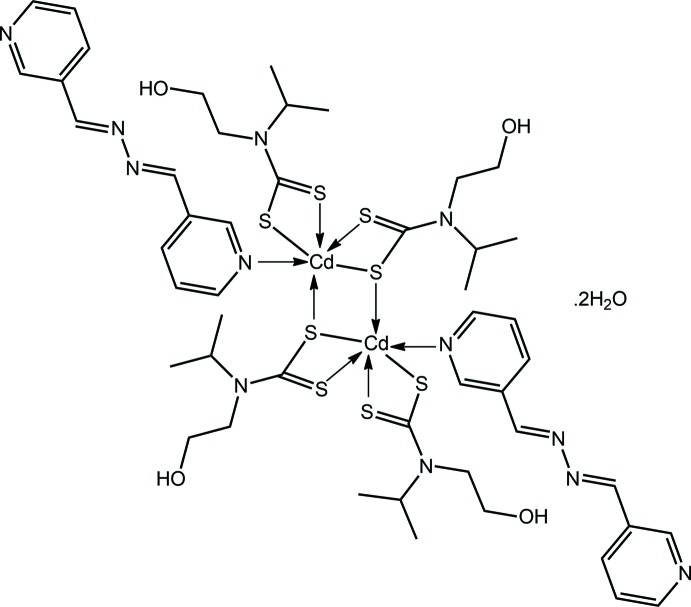


## Structural commentary   

**Figure 1:**
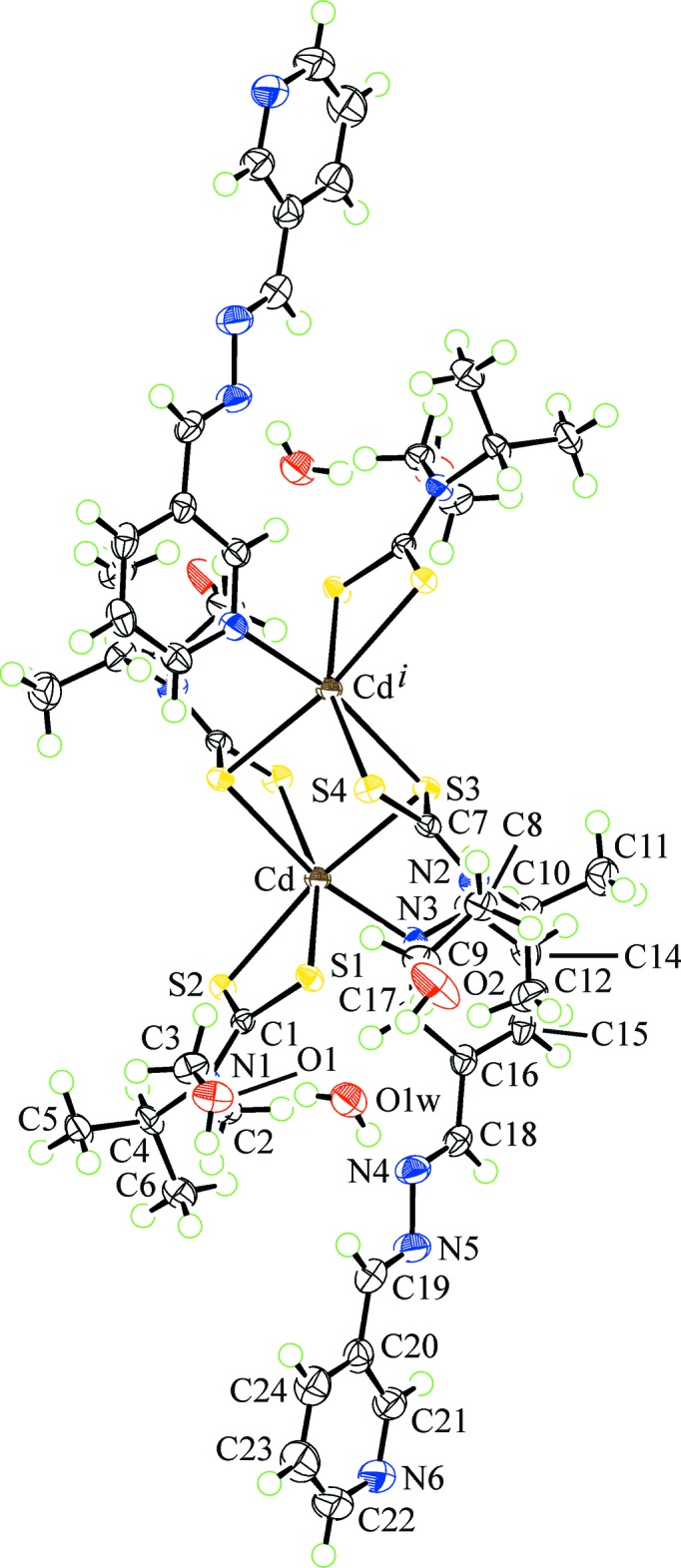
The mol­ecular structure of the binuclear title compound, showing the atom-labelling scheme and displacement ellipsoids at the 50% probability level. [Symmetry code: (i) 1 − *x*, −*y*, 1 − *z*.]

The mol­ecular structure of the binuclear title compound, isolated as a dihydrate, is shown in Fig. 1 ▸ and selected geometric parameters are collated in Table 1 ▸. The binuclear compound is disposed about a centre of inversion so the asymmetric comprises a Cd[S_2_CN(iPr)CH_2_CH_2_OH)]_2_ entity, a 3-pyridine­aldazine ligand and one water mol­ecule of solvation. One di­thio­carbamate (dtc) ligand coordinates in a chelating mode forming very similar Cd—S bond lengths, *i.e*. the difference between the Cd—S_short_ and Cd—S_long_ bond lengths is only 0.033 Å; this equivalence is reflected in the equivalence in the associated C1—S1, S2 bond lengths, Table 1 ▸. The second independent dtc chelates one cadmium atom and at the same time bridges the other cadmium atom. The Cd—S3_bridging_ bond lengths are close to being equal, differing by only 0.010 Å, and are longer by *ca* 0.1 Å than the non-bridging Cd—S4 bond length, Table 1 ▸. The differences in the number and strength of the Cd—S bond lengths for the S3-dtc ligand is reflected in the C7—S3, S4 separations with the C7—S4 bond length of 1.714 (2) Å being the shortest across the series. The sixth position in the distorted octa­hedral coordination geometry is occupied by a nitro­gen atom of the monodentate 3-pyridine­aldazine ligand. Distortions in angles about the cadmium atom are largely related to the restricted bite distances of the dtc ligands, Table 1 ▸. While not having crystallographic symmetry, the 3-pyridine­aldazine mol­ecule adopts an *anti* disposition about both imine bonds, *i.e*. C18=N4 = 1.283 (3) Å and C19=N5 = 1.277 (3) Å; the central, azo bond is 1.415 (2) Å. The pyridyl-N atoms are also *anti* but there are twists in the 3-pyridine­aldazine mol­ecule, as seen in the value of the dihedral angle between the two pyridyl rings of 22.78 (12)°.

## Supra­molecular features   

**Figure 2:**
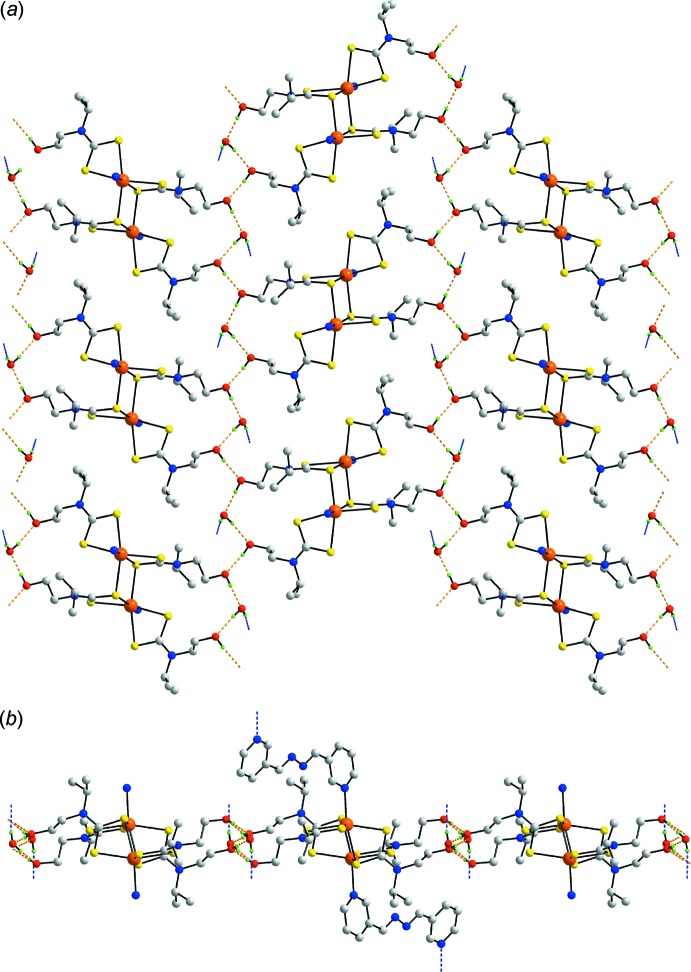
Mol­ecular packing: (*a*) view of the supra­molecular layer sustained by hy­droxy-O—H⋯O(hy­droxy) and hy­droxy-O—H⋯O(water) hydrogen bonds, shown as orange dashed lines. Only the pyridyl N atoms of the 3-pyridine­aldazine ligands are shown. (*b*) A side-on view of the layer in (*a*) extended to show the two central 3-pyridine­aldazine ligands (see text). The putative water-O—H⋯N(pyrid­yl) hydrogen bonds are shown as blue dashed lines. In both images, only acidic hydrogen atoms are included.

**Figure 3:**
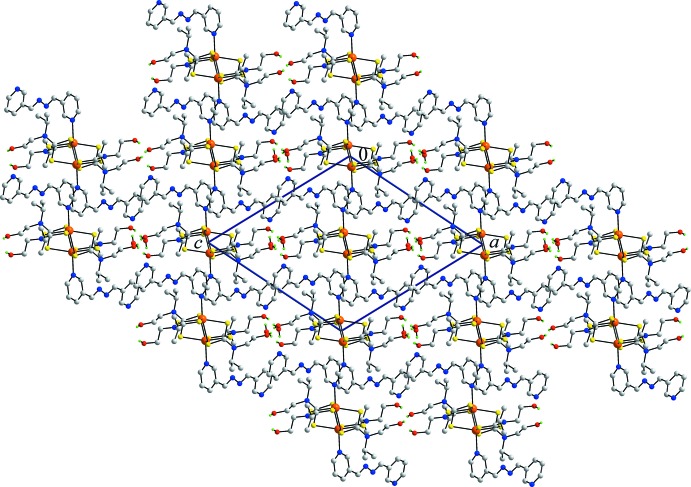
A view of the unit cell contents shown in projection down the *b* axis, highlighting the alternating layers of {Cd[S_2_CN(iPr)CH_2_CH_2_OH)]_2_}_2_ and 3-pyridine­aldazine mol­ecules. Water mol­ecules are located in the hy­droxy-rich regions, *i.e*. the key inter­faces between layers.

Significant O—H⋯O hydrogen bonding is found in the mol­ecular packing of the binuclear title compound as would be expected from the chemical composition. Thus, mol­ecules are assembled into layers approximately parallel to (101) by hy­droxy-O—H⋯O(hydrox­yl) and hy­droxy-O—H⋯O(water) hydrogen bonds as detailed in Table 2 ▸. Thus, strings of {⋯O_hy­droxy_—H⋯O_hy­droxy_—H⋯O_water_—H}_*n*_ chains are formed as shown in Fig. 2 ▸ *a*. The water mol­ecules also form water-O—H⋯N(pyrid­yl) hydrogen bonds on either side of the supra­molecular layers sustained by O—H⋯O hydrogen bonds, Fig. 2 ▸ *b*. The pendent pyridyl-N atoms of Fig. 2 ▸ *b* are coordinating to cadmium atoms of successive layers so that a three-dimensional architecture results. Globally, and as seen from Fig. 3 ▸, the mol­ecular packing comprises alternating layers of {Cd[S_2_CN(iPr)CH_2_CH_2_OH)]_2_}_2_ and 3-pyridine­aldazine with the key links between them being hydrogen and coordinate bonding. Within this framework stabilized primarily by hydrogen-bonding inter­actions, there are some second tier inter­actions worthy of comment (Spek, 2009 ▸). Thus, referring to data in Table 2 ▸, the hydroxyl-O1 atom also accepts a contact from a pyridyl-C—H atom as the 3-pyridine­aldazine ligand is orientated so that the non-coord­inating end is directed over the hy­droxy/water-rich region of the structure. Within the layers shown in Fig. 2 ▸ *a*, methine-C—H⋯S inter­actions are seen and between layers pyridyl-C—H⋯S contacts, inter­estingly, both involving the S2 atom. Finally, as has increasingly been noted in recent descriptions of the structural chemistry of metal di­thio­carbamates, C—H⋯π(chelate) inter­actions are present (Tiekink & Zukerman-Schpector, 2011 ▸). Here, a pyridyl-C—H atom sits almost perpendicular to the chelate ring involving the S1-di­thio­carbamate ligand, *i.e*. the C—H⋯ring centroid(chelate ring) angle is 178°, in the inter-layer region, Table 2 ▸.

## Database survey   

There are 14 examples of cadmium compounds with 3-pyridine­aldazine in the crystallographic literature (Groom *et al.*, 2016 ▸). Most of these feature μ_2_-bridging 3-pyridine­aldazine such as in the two most relevant compounds to the present study, namely {Cd[S_2_P(O-iPr)_2_]_2_(μ_2_-3-pyridine­aldazine)}_*n*_ (Lai & Tiekink, 2006*a* ▸) and bulky analogue {Cd[S_2_P(O-cHex)_2_]_2_(μ_2_-3-pyridine­aldazine)}_*n*_ (Lai & Tiekink, 2006*b* ▸). In the structure of {Cd[O_2_P(O-tBu)_2_]_2_(3-pyridine­aldazine)_2_(μ_2_-3-pyridine­aldazine)·H_2_O}_*n*_ (Rajakannu *et al.*, 2015 ▸), both bridging and monodentate 3-pyridine­aldazine ligands, in a 1:2 ratio, are observed. Underscoring the flexibility in mode of association of 3-pyridine­aldazine in their crystal structures, in {[Cd(3-pyridine­aldazine)_2_(μ_2_-3-pyridine­aldazine)(OH_2_)_2_](3-pyridine­aldazine)·2ClO_4_}_*n*_ (Bhattacharya *et al.*, 2013 ▸), bridging, monodentate and non-coordinating 3-pyridine­aldazine ligands, in a 1:2:1 ratio, are noted.

The most curious feature of the structure of the title compound is the retention of the central binuclear core. This is unprecedented in the structural chemistry of cadmium di­thio­carbamates (see *Chemical context*). A good number of zinc and mercury binary di­thio­carbamates are also known to adopt related binuclear [*M*(S_2_CN*RR*’)_2_]_2_ aggregates owing to the presence of equal numbers of μ_2_-tridentate and chelating ligands (Tiekink, 2003 ▸; Jotani *et al.*, 2016 ▸). Without exception, these are broken down upon adduct formation, regardless of the nature of the donor atom(s) (Groom *et al.*, 2016 ▸). This makes more curious the recent report of the mol­ecular structure of a cadmium xanthate adduct, [Cd(S_2_CO-iPr)_2_(hmta)]_2_, where hmta is hexa­methyl­ene­tetra­mine, for which an analogous centrosymmetric core and NS_5_ donor set as in the title compound was observed (Tan, Azizuddin *et al.*, 2016 ▸). This is quite unusual as there are no precedents for such [Cd(S_2_CO*R*)_2_]_2_ cores in the structural chemistry of metal xanthates (Tiekink & Haiduc, 2005 ▸). Clearly, as more study continues in this field, more inter­esting outcomes will be noted and rationalizations emerge.

## Synthesis and crystallization   

Cd[S_2_CN(iPr)CH_2_CH_2_OH)]_2_ (235 mg, 0.5 mmol) and 3-pyridine­aldazine (110 mg, 0.5 mmol) were dissolved in 1-propanol (15 ml). The solution was carefully covered with hexa­nes. Yellow blocks were obtained *via* slow diffusion of hexa­nes into the 1-propanol solution over two weeks. m.p. 389–391 K. IR (cm^−1^): 1449 (*m*) ν(C=N), 1173 (*s*) ν(C—S). ^1^H NMR: δ 9.04 (*d*, Ar, *J* = 1.46 Hz), 8.81 (*s*, Ar), 8.72 (*d*, Ar, *J* = 1.46 Hz), 8.29 (*d*, Ar, *J* = 1.95 Hz), 7.56 (*qd*, HC=CH, *J* = 4.88 Hz), 5.22 [sept, CH(CH_3_)_2_, *J* = 6.83 Hz], 4.83 (*t*, OH, *J* = 5.37 Hz), 3.74 (*d*, CH_2_O, *J* = 6.83 Hz), 3.68 (*d*, NCH_2_, *J* = 6.83 Hz), 1.18 (*d*, CH_3_, *J* = 6.84 Hz). TGA: three steps, corresponding to loss of water (calculated weight loss 2.6%; experimental weight loss 2.3%; onset 352 K, mid-point 364 K, endset 378 K), loss of the 3-pyridine­aldazine ligand (calculated weight loss 30.2%; experimental weight loss 30.5%; onset 418 K, mid-point 496 K, endset 511 K), and decomposition down to cadmium sulfide (calculated weight loss 79.3; experimental weight loss 75.1%; onset 542 K, mid-point 576 K, endset 620 K).

## Refinement   

Crystal data, data collection and structure refinement details are summarized in Table 3 ▸. The carbon-bound H atoms were placed in calculated positions (C—H = 0.95–1.00 Å) and were included in the refinement in the riding model approximation, with *U*_iso_(H) set at 1.2–1.5*U*_eq_(C). The oxygen-bound H atoms were located in a difference Fourier map but were refined with a distance restraint of O—H = 0.84±0.01 Å, and with *U*_iso_(H) set at 1.5*U*_eq_(O).

## Supplementary Material

Crystal structure: contains datablock(s) I, global. DOI: 10.1107/S2056989016012214/wm5312sup1.cif

Structure factors: contains datablock(s) I. DOI: 10.1107/S2056989016012214/wm5312Isup2.hkl

CCDC reference: 1496352

Additional supporting information: crystallographic information; 3D view; checkCIF report

## Figures and Tables

**Table 1:** Selected geometric parameters (Å, °)

Cd—S1	2.6444 (5)	Cd—N3	2.3811 (18)
Cd—S2	2.6768 (5)	S1—C1	1.7267 (19)
Cd—S3	2.7422 (5)	S2—C1	1.7231 (18)
Cd—S3^i^	2.7317 (6)	S3—C7	1.7404 (19)
Cd—S4^i^	2.6342 (5)	S4—C7	1.714 (2)
			
S1—Cd—S2	67.824 (14)	S2—Cd—S3	167.393 (15)
S4^i^—Cd—S3^i^	67.343 (17)	N3—Cd—S3^i^	166.35 (4)
S4^i^—Cd—S1	160.481 (17)		

Symmetry code: (i) 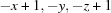.

**Table 2:** Hydrogen-bond geometry (Å, °)

*D*—H⋯*A*	*D*—H	H⋯*A*	*D*⋯*A*	*D*—H⋯*A*
O1—H1*O*⋯O2^ii^	0.83 (2)	1.83 (3)	2.655 (3)	172 (3)
O2—H2*O*⋯O1*W*	0.85 (3)	1.80 (3)	2.640 (3)	180 (6)
O1*W*—H1*W*⋯O1	0.84 (3)	1.92 (3)	2.750 (3)	172 (3)
O1*W*—H2*W*⋯N6^iii^	0.85 (2)	2.00 (2)	2.840 (3)	172 (2)
C23—H23⋯O1^ii^	0.95	2.50	3.295 (3)	141
C4—H4⋯S2^iv^	1.00	2.79	3.599 (2)	139
C15—H15⋯S2^v^	0.95	2.84	3.714 (2)	153
C15—H15⋯*Cg*(Cd,S1,S2,C1)^vi^	0.95	2.79	3.737 (2)	173

Symmetry codes: (ii) 
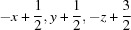
; (iii) 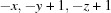; (iv) 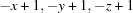; (v) 
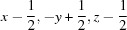
; (vi) 
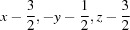
.

**Table 3:** Experimental details

Crystal data	
Chemical formula	[Cd(C_6_H_12_NOS_2_)_2_(C_12_H_10_N_4_)]_2_·2H_2_O
*M*_r_	1394.48
Crystal system, space group	Monoclinic, *P*2_1_/*n*
Temperature (K)	98
*a*, *b*, *c* (Å)	16.4700 (18), 12.2257 (12), 17.0862 (19)
β (°)	114.932 (2)
*V* (Å^3^)	3119.8 (6)
*Z*	2
Radiation type	Mo *K*α
μ (mm^−1^)	1.00
Crystal size (mm)	0.40 × 0.30 × 0.08

Data collection	
Diffractometer	AFC12K/SATURN724
Absorption correction	Multi-scan (*ABSCOR*; Higashi, 1995 ▸)
*T*_min_, *T*_max_	0.661, 1.000
No. of measured, independent and observed [*I* > 2σ(*I*)] reflections	22846, 7133, 6807
*R*_int_	0.029
(sin θ/λ)_max_ (Å^−1^)	0.650

Refinement	
*R*[*F* ^2^ > 2σ(*F* ^2^)], *wR*(*F* ^2^), *S*	0.029, 0.065, 1.08
No. of reflections	7133
No. of parameters	359
No. of restraints	4
Δρ_max_, Δρ_min_ (e Å^−3^)	0.54, −0.39

Computer programs: *CrystalClear* (Molecular Structure Corporation & Rigaku, 2005 ▸), *SHELXS97* (Sheldrick, 2008 ▸), *SHELXL2014* (Sheldrick, 2015 ▸), *ORTEP-3 for Windows* (Farrugia, 2012 ▸), *DIAMOND* (Brandenburg, 2006 ▸) and *publCIF* (Westrip, 2010 ▸).

## supplementary crystallographic information

### Crystal data

**Table:**

[Cd(C_6_H_12_NOS_2_)_2_(C_12_H_10_N_4_)_2_]_2_·2H_2_O	*F*(000) = 1432
*M**_r_* = 1394.48	*D*_x_ = 1.484 Mg m^−^^3^
Monoclinic, *P*2_1_/*n*	Mo *K*α radiation, λ = 0.71073 Å
*a* = 16.4700 (18) Å	Cell parameters from 19902 reflections
*b* = 12.2257 (12) Å	θ = 2.7–40.8°
*c* = 17.0862 (19) Å	µ = 1.00 mm^−^^1^
β = 114.932 (2)°	*T* = 98 K
*V* = 3119.8 (6) Å^3^	Plate, yellow
*Z* = 2	0.40 × 0.30 × 0.08 mm

### Data collection

**Table:**

AFC12K/SATURN724 diffractometer	7133 independent reflections
Radiation source: fine-focus sealed tube	6807 reflections with *I* > 2σ(*I*)
Graphite monochromator	*R*_int_ = 0.029
ω scans	θ_max_ = 27.5°, θ_min_ = 2.7°
Absorption correction: multi-scan (ABSCOR; Higashi, 1995)	*h* = −20→21
*T*_min_ = 0.661, *T*_max_ = 1.000	*k* = −15→15
22846 measured reflections	*l* = −22→19

### Refinement

**Table:**

Refinement on *F*^2^	4 restraints
Least-squares matrix: full	Hydrogen site location: mixed
*R*[*F*^2^ > 2σ(*F*^2^)] = 0.029	*w* = 1/[σ^2^(*F*_o_^2^) + (0.0243*P*)^2^ + 2.9797*P*] where *P* = (*F*_o_^2^ + 2*F*_c_^2^)/3
*wR*(*F*^2^) = 0.065	(Δ/σ)_max_ = 0.001
*S* = 1.08	Δρ_max_ = 0.54 e Å^−^^3^
7133 reflections	Δρ_min_ = −0.39 e Å^−^^3^
359 parameters	

### Special details

**Table:**

Geometry. All esds (except the esd in the dihedral angle between two l.s. planes) are estimated using the full covariance matrix. The cell esds are taken into account individually in the estimation of esds in distances, angles and torsion angles; correlations between esds in cell parameters are only used when they are defined by crystal symmetry. An approximate (isotropic) treatment of cell esds is used for estimating esds involving l.s. planes.

### Fractional atomic coordinates and isotropic or equivalent isotropic displacement parameters (Å^2^)

**Table:**

	*x*	*y*	*z*	*U*_iso_*/*U*_eq_	
Cd	0.43075 (2)	0.13535 (2)	0.44655 (2)	0.01368 (5)	
S1	0.36321 (3)	0.19066 (4)	0.55658 (3)	0.01568 (10)	
S2	0.43474 (3)	0.35177 (4)	0.47165 (3)	0.01378 (9)	
S3	0.39975 (3)	−0.08571 (4)	0.43612 (3)	0.01393 (9)	
S4	0.45562 (3)	−0.11726 (4)	0.62449 (3)	0.01652 (10)	
O1	0.36420 (11)	0.30641 (13)	0.79528 (10)	0.0241 (3)	
H1O	0.3348 (17)	0.3606 (15)	0.797 (2)	0.036*	
O2	0.24285 (16)	−0.03206 (14)	0.70325 (14)	0.0425 (5)	
H2O	0.241 (3)	0.0271 (17)	0.728 (2)	0.064*	
O1W	0.23612 (12)	0.15309 (13)	0.77985 (12)	0.0276 (4)	
H1W	0.2791 (15)	0.195 (2)	0.787 (2)	0.041*	
H2W	0.1898 (13)	0.185 (2)	0.7437 (16)	0.041*	
N1	0.28639 (11)	−0.08842 (14)	0.51175 (11)	0.0165 (3)	
N2	0.37924 (11)	0.40314 (13)	0.59396 (11)	0.0150 (3)	
N3	0.29195 (12)	0.15099 (13)	0.32251 (11)	0.0177 (3)	
N4	0.11563 (13)	0.38202 (15)	0.34081 (13)	0.0242 (4)	
N5	0.05594 (13)	0.46819 (16)	0.33476 (13)	0.0260 (4)	
N6	−0.08839 (14)	0.74203 (17)	0.35311 (15)	0.0324 (5)	
C1	0.39084 (12)	0.32403 (15)	0.54493 (12)	0.0126 (3)	
C2	0.33799 (13)	0.37919 (16)	0.65353 (13)	0.0165 (4)	
H2A	0.2888	0.3259	0.6262	0.020*	
H2B	0.3119	0.4472	0.6646	0.020*	
C3	0.40587 (15)	0.33254 (18)	0.73950 (14)	0.0218 (4)	
H3A	0.4335	0.2658	0.7284	0.026*	
H3B	0.4540	0.3868	0.7680	0.026*	
C4	0.40109 (14)	0.51947 (15)	0.58531 (14)	0.0178 (4)	
H4	0.4361	0.5203	0.5497	0.021*	
C5	0.46023 (15)	0.56990 (17)	0.67257 (15)	0.0227 (4)	
H5A	0.5143	0.5255	0.7010	0.034*	
H5B	0.4273	0.5723	0.7087	0.034*	
H5C	0.4771	0.6443	0.6640	0.034*	
C6	0.31638 (15)	0.58567 (17)	0.53696 (15)	0.0238 (4)	
H6A	0.2798	0.5495	0.4821	0.036*	
H6B	0.3325	0.6592	0.5256	0.036*	
H6C	0.2823	0.5909	0.5720	0.036*	
C7	0.37201 (13)	−0.09619 (15)	0.52349 (13)	0.0144 (4)	
C8	0.26157 (15)	−0.10857 (17)	0.58411 (14)	0.0198 (4)	
H8A	0.2988	−0.1687	0.6203	0.024*	
H8B	0.1982	−0.1319	0.5609	0.024*	
C9	0.27426 (16)	−0.00701 (17)	0.63969 (15)	0.0237 (4)	
H9A	0.3383	0.0135	0.6676	0.028*	
H9B	0.2400	0.0551	0.6037	0.028*	
C10	0.21194 (14)	−0.07222 (17)	0.42431 (14)	0.0207 (4)	
H10	0.2392	−0.0431	0.3861	0.025*	
C11	0.16893 (16)	−0.1815 (2)	0.38682 (16)	0.0305 (5)	
H11A	0.2142	−0.2307	0.3832	0.046*	
H11B	0.1211	−0.1701	0.3290	0.046*	
H11C	0.1438	−0.2141	0.4241	0.046*	
C12	0.14460 (15)	0.0123 (2)	0.42532 (17)	0.0299 (5)	
H12A	0.1756	0.0812	0.4491	0.045*	
H12B	0.1154	−0.0143	0.4612	0.045*	
H12C	0.0994	0.0245	0.3663	0.045*	
C13	0.26289 (15)	0.08493 (17)	0.25306 (14)	0.0201 (4)	
H13	0.3011	0.0281	0.2507	0.024*	
C14	0.17944 (15)	0.09657 (18)	0.18503 (14)	0.0227 (4)	
H14	0.1606	0.0482	0.1371	0.027*	
C15	0.12347 (14)	0.18035 (17)	0.18798 (14)	0.0209 (4)	
H15	0.0665	0.1912	0.1414	0.025*	
C16	0.15226 (13)	0.24779 (16)	0.26003 (13)	0.0170 (4)	
C17	0.23735 (14)	0.22991 (16)	0.32592 (13)	0.0180 (4)	
H17	0.2573	0.2758	0.3753	0.022*	
C18	0.09590 (14)	0.33688 (17)	0.26722 (14)	0.0199 (4)	
H18	0.0452	0.3609	0.2178	0.024*	
C19	0.07322 (14)	0.50864 (18)	0.40903 (15)	0.0234 (4)	
H19	0.1193	0.4773	0.4588	0.028*	
C20	0.02300 (15)	0.60274 (18)	0.41864 (15)	0.0231 (4)	
C21	−0.04395 (15)	0.65334 (19)	0.34710 (16)	0.0256 (5)	
H21	−0.0585	0.6232	0.2915	0.031*	
C22	−0.06761 (18)	0.7822 (2)	0.4324 (2)	0.0400 (6)	
H22	−0.0991	0.8450	0.4374	0.048*	
C23	−0.00341 (19)	0.7380 (2)	0.50682 (18)	0.0378 (6)	
H23	0.0087	0.7693	0.5615	0.045*	
C24	0.04320 (17)	0.6467 (2)	0.50016 (17)	0.0301 (5)	
H24	0.0882	0.6145	0.5503	0.036*	

### Atomic displacement parameters (Å^2^)

**Table:**

	*U*^11^	*U*^22^	*U*^33^	*U*^12^	*U*^13^	*U*^23^
Cd	0.01391 (8)	0.01315 (7)	0.01351 (8)	0.00222 (5)	0.00533 (6)	−0.00025 (5)
S1	0.0203 (2)	0.0118 (2)	0.0179 (2)	−0.00160 (17)	0.0110 (2)	−0.00003 (17)
S2	0.0168 (2)	0.0131 (2)	0.0133 (2)	−0.00056 (17)	0.00812 (18)	−0.00001 (16)
S3	0.0158 (2)	0.0136 (2)	0.0135 (2)	0.00129 (17)	0.00717 (18)	0.00009 (16)
S4	0.0174 (2)	0.0196 (2)	0.0135 (2)	0.00195 (18)	0.00735 (19)	0.00139 (18)
O1	0.0322 (9)	0.0246 (8)	0.0226 (8)	0.0050 (7)	0.0187 (7)	0.0051 (6)
O2	0.0775 (15)	0.0232 (8)	0.0574 (13)	−0.0139 (9)	0.0583 (13)	−0.0092 (8)
O1W	0.0316 (9)	0.0233 (8)	0.0317 (10)	−0.0008 (7)	0.0172 (8)	−0.0012 (7)
N1	0.0162 (8)	0.0177 (8)	0.0177 (9)	0.0016 (6)	0.0091 (7)	0.0033 (6)
N2	0.0183 (8)	0.0129 (7)	0.0165 (8)	−0.0012 (6)	0.0100 (7)	−0.0009 (6)
N3	0.0198 (8)	0.0160 (8)	0.0145 (8)	0.0015 (7)	0.0044 (7)	−0.0003 (6)
N4	0.0212 (9)	0.0250 (9)	0.0234 (10)	0.0069 (7)	0.0065 (8)	0.0021 (7)
N5	0.0240 (9)	0.0266 (9)	0.0262 (10)	0.0069 (8)	0.0094 (8)	0.0004 (8)
N6	0.0272 (10)	0.0331 (11)	0.0339 (12)	0.0045 (9)	0.0099 (9)	−0.0078 (9)
C1	0.0116 (8)	0.0149 (8)	0.0105 (9)	0.0001 (7)	0.0038 (7)	−0.0003 (7)
C2	0.0178 (9)	0.0181 (9)	0.0172 (10)	0.0006 (7)	0.0110 (8)	−0.0005 (7)
C3	0.0227 (10)	0.0273 (10)	0.0182 (10)	0.0028 (9)	0.0114 (9)	0.0017 (8)
C4	0.0238 (10)	0.0122 (8)	0.0209 (10)	−0.0034 (8)	0.0127 (9)	−0.0024 (7)
C5	0.0266 (11)	0.0169 (9)	0.0247 (11)	−0.0049 (8)	0.0111 (9)	−0.0067 (8)
C6	0.0291 (11)	0.0187 (10)	0.0236 (11)	0.0012 (9)	0.0112 (10)	0.0011 (8)
C7	0.0169 (9)	0.0103 (8)	0.0167 (9)	0.0006 (7)	0.0077 (8)	0.0008 (7)
C8	0.0225 (10)	0.0198 (9)	0.0232 (11)	−0.0011 (8)	0.0155 (9)	0.0016 (8)
C9	0.0310 (11)	0.0208 (10)	0.0301 (12)	0.0005 (9)	0.0232 (10)	0.0020 (9)
C10	0.0156 (9)	0.0241 (10)	0.0210 (11)	0.0016 (8)	0.0064 (8)	0.0035 (8)
C11	0.0277 (12)	0.0285 (12)	0.0278 (13)	−0.0016 (10)	0.0045 (10)	−0.0011 (10)
C12	0.0213 (11)	0.0304 (12)	0.0351 (14)	0.0054 (9)	0.0092 (10)	0.0033 (10)
C13	0.0258 (11)	0.0168 (9)	0.0167 (10)	0.0008 (8)	0.0080 (9)	−0.0008 (8)
C14	0.0294 (11)	0.0223 (10)	0.0141 (10)	−0.0051 (9)	0.0069 (9)	−0.0018 (8)
C15	0.0184 (10)	0.0232 (10)	0.0162 (10)	−0.0042 (8)	0.0026 (8)	0.0029 (8)
C16	0.0164 (9)	0.0163 (9)	0.0175 (10)	−0.0026 (7)	0.0064 (8)	0.0029 (7)
C17	0.0194 (10)	0.0156 (9)	0.0168 (10)	0.0003 (8)	0.0056 (8)	−0.0003 (7)
C18	0.0151 (9)	0.0218 (10)	0.0206 (10)	0.0002 (8)	0.0052 (8)	0.0051 (8)
C19	0.0181 (10)	0.0280 (11)	0.0227 (11)	−0.0007 (8)	0.0073 (9)	0.0030 (9)
C20	0.0209 (10)	0.0259 (10)	0.0247 (11)	−0.0042 (9)	0.0118 (9)	−0.0028 (9)
C21	0.0231 (11)	0.0296 (11)	0.0234 (12)	0.0015 (9)	0.0091 (9)	−0.0041 (9)
C22	0.0339 (14)	0.0411 (14)	0.0447 (16)	0.0055 (12)	0.0162 (13)	−0.0165 (13)
C23	0.0384 (14)	0.0480 (15)	0.0295 (14)	−0.0030 (12)	0.0167 (12)	−0.0160 (12)
C24	0.0289 (12)	0.0369 (13)	0.0246 (12)	−0.0019 (10)	0.0113 (10)	−0.0019 (10)

### Geometric parameters (Å, º)

**Table:**

Cd—S1	2.6444 (5)	C5—H5B	0.9800
Cd—S2	2.6768 (5)	C5—H5C	0.9800
Cd—S3	2.7422 (5)	C6—H6A	0.9800
Cd—S3^i^	2.7317 (6)	C6—H6B	0.9800
Cd—S4^i^	2.6342 (5)	C6—H6C	0.9800
Cd—N3	2.3811 (18)	C8—C9	1.523 (3)
S1—C1	1.7267 (19)	C8—H8A	0.9900
S2—C1	1.7231 (18)	C8—H8B	0.9900
S3—C7	1.7404 (19)	C9—H9A	0.9900
S3—Cd^i^	2.7317 (6)	C9—H9B	0.9900
S4—C7	1.714 (2)	C10—C11	1.521 (3)
S4—Cd^i^	2.6343 (5)	C10—C12	1.521 (3)
O1—C3	1.426 (2)	C10—H10	1.0000
O1—H1O	0.830 (10)	C11—H11A	0.9800
O2—C9	1.419 (2)	C11—H11B	0.9800
O2—H2O	0.846 (10)	C11—H11C	0.9800
O1W—H1W	0.842 (10)	C12—H12A	0.9800
O1W—H2W	0.847 (10)	C12—H12B	0.9800
N1—C7	1.341 (2)	C12—H12C	0.9800
N1—C8	1.477 (2)	C13—C14	1.383 (3)
N1—C10	1.493 (3)	C13—H13	0.9500
N2—C1	1.345 (2)	C14—C15	1.393 (3)
N2—C2	1.472 (2)	C14—H14	0.9500
N2—C4	1.490 (2)	C15—C16	1.388 (3)
N3—C17	1.337 (3)	C15—H15	0.9500
N3—C13	1.346 (3)	C16—C17	1.396 (3)
N4—C18	1.283 (3)	C16—C18	1.469 (3)
N4—N5	1.415 (2)	C17—H17	0.9500
N5—C19	1.277 (3)	C18—H18	0.9500
N6—C21	1.336 (3)	C19—C20	1.466 (3)
N6—C22	1.342 (3)	C19—H19	0.9500
C2—C3	1.532 (3)	C20—C24	1.396 (3)
C2—H2A	0.9900	C20—C21	1.399 (3)
C2—H2B	0.9900	C21—H21	0.9500
C3—H3A	0.9900	C22—C23	1.377 (4)
C3—H3B	0.9900	C22—H22	0.9500
C4—C6	1.521 (3)	C23—C24	1.386 (4)
C4—C5	1.526 (3)	C23—H23	0.9500
C4—H4	1.0000	C24—H24	0.9500
C5—H5A	0.9800		
			
N3—Cd—S4^i^	101.46 (4)	N1—C7—S4	120.60 (15)
N3—Cd—S1	94.50 (4)	N1—C7—S3	120.40 (15)
N3—Cd—S2	90.73 (4)	S4—C7—S3	119.00 (11)
S4^i^—Cd—S2	100.522 (15)	N1—C8—C9	111.86 (16)
S1—Cd—S2	67.824 (14)	N1—C8—H8A	109.2
S4^i^—Cd—S3^i^	67.343 (17)	C9—C8—H8A	109.2
S4^i^—Cd—S1	160.481 (17)	N1—C8—H8B	109.2
S2—Cd—S3	167.393 (15)	C9—C8—H8B	109.2
N3—Cd—S3^i^	166.35 (4)	H8A—C8—H8B	107.9
S1—Cd—S3^i^	98.110 (17)	O2—C9—C8	107.62 (17)
S2—Cd—S3^i^	98.831 (15)	O2—C9—H9A	110.2
N3—Cd—S3	86.49 (4)	C8—C9—H9A	110.2
S4^i^—Cd—S3	92.082 (15)	O2—C9—H9B	110.2
S1—Cd—S3	100.113 (14)	C8—C9—H9B	110.2
S3^i^—Cd—S3	86.184 (15)	H9A—C9—H9B	108.5
C1—S1—Cd	87.09 (6)	N1—C10—C11	110.18 (17)
C1—S2—Cd	86.13 (6)	N1—C10—C12	111.95 (18)
C7—S3—Cd^i^	84.87 (7)	C11—C10—C12	112.89 (19)
C7—S3—Cd	97.23 (6)	N1—C10—H10	107.2
Cd^i^—S3—Cd	93.816 (15)	C11—C10—H10	107.2
C7—S4—Cd^i^	88.51 (7)	C12—C10—H10	107.2
C3—O1—H1O	108 (2)	C10—C11—H11A	109.5
C9—O2—H2O	107 (3)	C10—C11—H11B	109.5
H1W—O1W—H2W	106 (3)	H11A—C11—H11B	109.5
C7—N1—C8	120.49 (17)	C10—C11—H11C	109.5
C7—N1—C10	121.81 (16)	H11A—C11—H11C	109.5
C8—N1—C10	117.33 (16)	H11B—C11—H11C	109.5
C1—N2—C2	121.05 (16)	C10—C12—H12A	109.5
C1—N2—C4	121.41 (16)	C10—C12—H12B	109.5
C2—N2—C4	117.38 (15)	H12A—C12—H12B	109.5
C17—N3—C13	118.42 (18)	C10—C12—H12C	109.5
C17—N3—Cd	115.48 (14)	H12A—C12—H12C	109.5
C13—N3—Cd	126.01 (14)	H12B—C12—H12C	109.5
C18—N4—N5	111.56 (18)	N3—C13—C14	122.51 (19)
C19—N5—N4	111.02 (19)	N3—C13—H13	118.7
C21—N6—C22	117.4 (2)	C14—C13—H13	118.7
N2—C1—S2	121.63 (14)	C13—C14—C15	118.9 (2)
N2—C1—S1	119.59 (14)	C13—C14—H14	120.5
S2—C1—S1	118.77 (11)	C15—C14—H14	120.5
N2—C2—C3	111.96 (16)	C16—C15—C14	119.0 (2)
N2—C2—H2A	109.2	C16—C15—H15	120.5
C3—C2—H2A	109.2	C14—C15—H15	120.5
N2—C2—H2B	109.2	C15—C16—C17	118.27 (19)
C3—C2—H2B	109.2	C15—C16—C18	121.51 (19)
H2A—C2—H2B	107.9	C17—C16—C18	120.22 (19)
O1—C3—C2	111.22 (17)	N3—C17—C16	122.88 (19)
O1—C3—H3A	109.4	N3—C17—H17	118.6
C2—C3—H3A	109.4	C16—C17—H17	118.6
O1—C3—H3B	109.4	N4—C18—C16	119.52 (19)
C2—C3—H3B	109.4	N4—C18—H18	120.2
H3A—C3—H3B	108.0	C16—C18—H18	120.2
N2—C4—C6	110.94 (17)	N5—C19—C20	121.0 (2)
N2—C4—C5	111.74 (17)	N5—C19—H19	119.5
C6—C4—C5	112.17 (17)	C20—C19—H19	119.5
N2—C4—H4	107.2	C24—C20—C21	118.1 (2)
C6—C4—H4	107.2	C24—C20—C19	120.3 (2)
C5—C4—H4	107.2	C21—C20—C19	121.6 (2)
C4—C5—H5A	109.5	N6—C21—C20	123.2 (2)
C4—C5—H5B	109.5	N6—C21—H21	118.4
H5A—C5—H5B	109.5	C20—C21—H21	118.4
C4—C5—H5C	109.5	N6—C22—C23	123.9 (2)
H5A—C5—H5C	109.5	N6—C22—H22	118.0
H5B—C5—H5C	109.5	C23—C22—H22	118.0
C4—C6—H6A	109.5	C22—C23—C24	118.5 (2)
C4—C6—H6B	109.5	C22—C23—H23	120.8
H6A—C6—H6B	109.5	C24—C23—H23	120.8
C4—C6—H6C	109.5	C23—C24—C20	119.0 (2)
H6A—C6—H6C	109.5	C23—C24—H24	120.5
H6B—C6—H6C	109.5	C20—C24—H24	120.5
			
C18—N4—N5—C19	176.5 (2)	C7—N1—C10—C11	96.7 (2)
C2—N2—C1—S2	177.27 (14)	C8—N1—C10—C11	−76.4 (2)
C4—N2—C1—S2	2.0 (3)	C7—N1—C10—C12	−136.83 (19)
C2—N2—C1—S1	−3.9 (3)	C8—N1—C10—C12	50.1 (2)
C4—N2—C1—S1	−179.22 (14)	C17—N3—C13—C14	0.8 (3)
Cd—S2—C1—N2	174.63 (16)	Cd—N3—C13—C14	177.07 (15)
Cd—S2—C1—S1	−4.16 (10)	N3—C13—C14—C15	0.6 (3)
Cd—S1—C1—N2	−174.61 (16)	C13—C14—C15—C16	−1.6 (3)
Cd—S1—C1—S2	4.21 (11)	C14—C15—C16—C17	1.3 (3)
C1—N2—C2—C3	82.7 (2)	C14—C15—C16—C18	−179.43 (18)
C4—N2—C2—C3	−101.9 (2)	C13—N3—C17—C16	−1.1 (3)
N2—C2—C3—O1	−178.05 (16)	Cd—N3—C17—C16	−177.75 (15)
C1—N2—C4—C6	104.3 (2)	C15—C16—C17—N3	0.0 (3)
C2—N2—C4—C6	−71.1 (2)	C18—C16—C17—N3	−179.23 (18)
C1—N2—C4—C5	−129.72 (19)	N5—N4—C18—C16	178.04 (17)
C2—N2—C4—C5	54.8 (2)	C15—C16—C18—N4	165.2 (2)
C8—N1—C7—S4	−6.0 (2)	C17—C16—C18—N4	−15.5 (3)
C10—N1—C7—S4	−178.81 (14)	N4—N5—C19—C20	176.96 (18)
C8—N1—C7—S3	173.68 (14)	N5—C19—C20—C24	−179.4 (2)
C10—N1—C7—S3	0.9 (3)	N5—C19—C20—C21	−1.6 (3)
Cd^i^—S4—C7—N1	174.52 (15)	C22—N6—C21—C20	−1.0 (4)
Cd^i^—S4—C7—S3	−5.15 (10)	C24—C20—C21—N6	0.8 (3)
Cd^i^—S3—C7—N1	−174.68 (15)	C19—C20—C21—N6	−177.0 (2)
Cd—S3—C7—N1	92.11 (15)	C21—N6—C22—C23	0.4 (4)
Cd^i^—S3—C7—S4	4.99 (10)	N6—C22—C23—C24	0.3 (5)
Cd—S3—C7—S4	−88.22 (11)	C22—C23—C24—C20	−0.4 (4)
C7—N1—C8—C9	84.0 (2)	C21—C20—C24—C23	−0.1 (3)
C10—N1—C8—C9	−102.9 (2)	C19—C20—C24—C23	177.8 (2)
N1—C8—C9—O2	175.92 (19)		

Symmetry code: (i) −*x*+1, −*y*, −*z*+1.

### Hydrogen-bond geometry (Å, º)

**Table:**

*D*—H···*A*	*D*—H	H···*A*	*D*···*A*	*D*—H···*A*
O1—H1*O*···O2^ii^	0.83 (2)	1.83 (3)	2.655 (3)	172 (3)
O2—H2*O*···O1*W*	0.85 (3)	1.80 (3)	2.640 (3)	180 (6)
O1*W*—H1*W*···O1	0.84 (3)	1.92 (3)	2.750 (3)	172 (3)
O1*W*—H2*W*···N6^iii^	0.85 (2)	2.00 (2)	2.840 (3)	172 (2)
C23—H23···O1^ii^	0.95	2.50	3.295 (3)	141
C4—H4···S2^iv^	1.00	2.79	3.599 (2)	139
C15—H15···S2^v^	0.95	2.84	3.714 (2)	153
C15—H15···*Cg*(Cd,S1,S2,C1)^vi^	0.95	2.79	3.737 (2)	173

Symmetry codes: (ii) −*x*+1/2, *y*+1/2, −*z*+3/2; (iii) −*x*, −*y*+1, −*z*+1; (iv) −*x*+1, −*y*+1, −*z*+1; (v) *x*−1/2, −*y*+1/2, *z*−1/2; (vi) *x*−3/2, −*y*−1/2, *z*−3/2.
